# BioWes-from design of experiment, through protocol to repository, control, standardization and back-tracking

**DOI:** 10.1186/s12938-016-0188-8

**Published:** 2016-07-15

**Authors:** Petr Cisar, Dmytro Soloviov, Antonin Barta, Jan Urban, Dalibor Stys

**Affiliations:** Faculty of Fisheries and Protection of Waters, Institute of Complex Systems, South Bohemian Research Center of Aquaculture and Biodiversity of Hydrocenoses, University of South Bohemia in České Budějovice, Zámek 136, 37333 Nove Hrady, Czech Republic

**Keywords:** Data management, Reproducibility, Sharing, Data processing, Experimental data, Metadata, Standardization

## Abstract

**Background:**

One of the main challenges in modern science is the amount of data produced by the experimental work; it is difficult to store, organize and share the scientific data and to extract the wealth of knowledge. Experimental method descriptions in scientific publications are often incomplete, which complicates experimental reproducibility. The proposed system was created in order to address these issues. It provides a solution for management of the experimental data and metadata to support the reproducibility.

**Implementation:**

The system is implemented as a repository for experiment descriptions and experimental data. It has three main entry points: desktop application for protocol design and data processing, web interface dedicated for protocol and data management, and web-based interface for mobile devices suitable for the field experiments. The functionality of desktop client can be extended using the custom plug-ins for data extraction and data processing. The system provides several methods to support experimental reproducibility: standardized terminology support, data and metadata at a single location, standardized protocol design or protocol evolution.

**Results and discussion:**

The system was tested in the framework of international infrastructure project AQUAEXCEL with five pilot installations at different institutes. The general testing in Tissue culture certified laboratory, Institute of complex systems and IFREMER verified the usability under different research infrastructures. The specific testing focused on the data processing modules and plug-ins demonstrated the modularity of the system for the specific conditions. The BioWes system represents experimental data as black box and therefore can handle any data type so as to provide broad usability for a variety of experiments and provide the data management infrastructure to improve the reproducibility and data sharing.

**Conclusions:**

The proposed system provides the tools for standard data management operations and extends the support by the standardization possibilities, protocol evolution with visualization features and modularity based on the data processing modules and device communication plug-ins. The software can be used at different organization levels: from a single researcher (to improve data organization) to research consortium through the central protocols management repository. Support from the protocol design until being shared with the standardization features helps to improve the reproducibility of research work. The platform provides support from experimental protocol design to cooperation using simple sharing.

## Background

We are living in an age of “Big data” [1], which is changing all areas of human-kind including science. One of the most important issues in experimental research is the reproducibility of experiments. Achenbach [2] describes the actual situation in the world science, which is measured and driven by the peer-review publishing process. The reproducibility and replicability of experiments is becoming more and more critical relative to the enormous number of scientific papers published nowadays [3]. The reproducibility is highly connected to the proper description of experimental conditions, which can influence the results of the experiment. The experimental protocol is not only the measurable conditions under which we perform our experiments but it is a complete set of information called experimental metadata. To clarify the concept of experimental metadata, we must start with the general definition of metadata. Source [4, 5] defines metadata as data about data. The concept of metadata itself may involve either structural metadata (metadata related primarily to the design and structure of the data structures—data about data containers) or the descriptive metadata (metadata related to the content description data—the contents of the content). In terms of description of experiments and their reproducibility, we must talk about structural metadata. Experimental metadata should include all information that is critical to be able to repeat or reproduce the experiment and to achieve similar results. Thus, defined group is able to describe only the experiment itself, however, it does not contain additional information that is critical to extract experimental metadata. As an example, a person is responsible for an experiment, the timestamp of the experiment, the incorporation experiment in specific research areas, etc. For this reason it is necessary to become familiar with the system implementation and describe experiments from different sites in different areas of biological experiments, and assemble categories of metadata that are needed for good description of the experiment—therefore representing experimental metadata. It is necessary to adjust the definition of experimental metadata. Experimental metadata is data about data via which captures all the information needed to repeat an experiment and to use the metadata for automatic information retrieval from the perspective of the owner of experimental data in terms of data sharing. “Biological meta data are gold” [6], therefore we should concentrate very carefully, not only on the data itself, but also on the description of the experiments.

Research is more and more a product of interdisciplinary cooperation, which requires the capacity for data sharing. One of the main issues of data sharing is the lack of the data and metadata management infrastructures and inconsistency in standardization [7, 8]. There are various data and metadata management systems, which covers different aspects of experimental work. Laboratory management systems [9] are designed to provide tools for laboratory resource management and can be specific to some description of a particular laboratory process (experimental work). This software is usually designed for a specific experimental work and particular data types. The description of the experiment is predefined and restricts modifications by the user to adapt the protocol. More closely related software for metadata management are systems based on the “e-notebook” [10, 11]. These software packages provide tools for description of the experimental work in the form of electronic log books. It is usually non-standardized electronic document with the possibility to describe the experimental work and attach experimental data. The weakness of these systems is the free form of the protocol design. The experimentalist logs the experimental work as it is obtained during the experiment. The support of data processing and evolution of the protocol is not provided The free form of experimental work description complicates data mining or re-application for other experiment. Another option for data management is a data sharing and processing platform [12]. The platform is mainly focused on the data, models and data processing modules. They provide the standardization for data exchange and searching tools. The system uses existing metadata about the experimental data or models, which is fully dependent on the users. If a user wants to use the system, he/she must adapt the metadata to the format of the platform; this can be time consuming and can also discourage sharing the data. None of the systems provide comprehensive tools that start with the experimental protocol design, continue through data and metadata processing and end with cooperative sharing.

The BioWes solution has been designed to support researchers from the design of the experimental protocol until the sharing, using the same structure of the metadata to minimize the effort needed for data and metadata management and with a focus on reproducibility of the experimental work.

We performed a survey in 2011 with 17 partners about experimental data and metadata management. More information about the survey can be found on line (http://www.biowes.org/survey —only available in Czech). The results of the survey clearly identified issues relative to the data management process. In general, the institutes have good procedures of the experimental protocol preparation (description of experiment), very simple solutions for data storage and missing data management tools. The experimental protocols are defined in in a description of the experimental conditions. The standardization (terminology) is used occasionally and terminology specific to the group is often used. The institutes use personal computers, portable disks or an allocated server directory for the data storage, but with no hierarchical or file name convention. The protocols and experimental data are not directly linked so as to provide complete information about the experiment. The cooperation between partners is accomplished by data transfer via web- based storage or email. The missing metadata and data management tools do not allow them to benefit from the experiments organization, searching, visualization or standardization, thus reducing experimental reproducibility.

This paper presents a software platform for the experimental data and metadata management, beginning with protocol design to the data sharing. The platform consists of several modules (local database, Protocol Manager, Web Interface, central database) which enable the user to customize it for the specific needs of a single researcher or a research consortium. The platform focusez on support of the researcher so as to provide the tools to improve the reproducibility, cooperation and data organization. The overall concept is based on an electronic Protocol, which contains complete information about the experiment (experimental data + experiment description) and enables the system to provide data processing modules, plugins for communication with measurement devices or direct usage of the standardization. The platform is based on the software implemented in Microsoft .NET Framework 4 - C# and PHP.

The platform has several pilot installations at different research institutes and is one of the tools for scientific cooperation in the international research infrastructure project AQUAEXCEL [13].

## Implementation

**Fig. 1:**
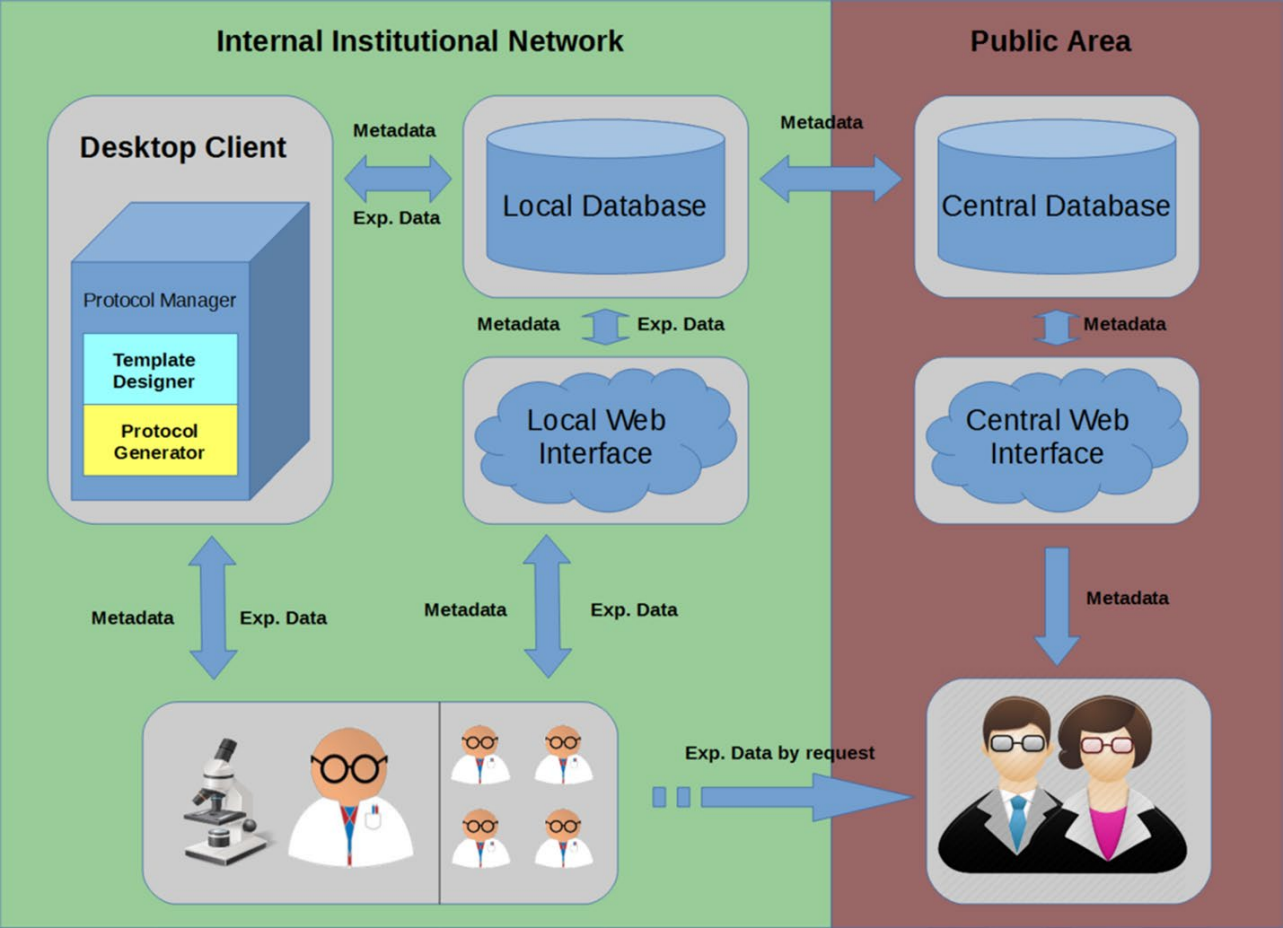
An overall overview of BioWes system

The BioWes platform consists of three main parts: desktop client, local repository and central repository as shown at Fig. 1. The desktop client is a software for the template design, protocol filling, communication with measurement devices and data processing. The local repository is based on the Microsoft SQL database that store all the information related to BioWes-protocols, templates, experimental data, user accounts, etc. at the local laboratory/institute/consortium member. The local repository is dedicated to the local usage only to secure sensitive experimental data and experiment descriptions. The central repository is also based on the Microsoft SQL. The interface to the central repository enables the public users to search for the specific experimental data based only on the description of experiment. No sensitive data are stored in the central repository. The description of experiments can be easily shared from the local repository to the central repository.

Three main entry points into the local repository running on a dedicated server in institution network are *desktop client*, *web interface* and *mobile interface*. Server with database, web Interface, mobile Interface and number of desktop clients connected to it represents an instance of BioWes on the level of single organization (lab, research institute, university, etc.).

Several instances of BioWes can be linked to the central repository with central database allowing collaboration and sharing among different organizations around the world. The main difference between central database and local database is that central database supports only metadata sharing. Sharing of experimental data is out of scope.

The desktop client and web interface communicates with the database using direct SQL queries. Protocols and Templates are sent and stored as XML files.

The general concept of the BioWes is based on the electronic protocol and black-box datasets. The electronic protocol has two stages: the first is the template which can be understood as empty protocol or experiment description without particular values. The second stage is the protocol (filled template) for a particular realization of the experiment. The scientific data is directly linked with the Protocol in the form of binary blocks. Therefore the system is able to store any datatype to support wide range of the experiments.

### Desktop client

Desktop client is a stand-alone program implemented in C# programming language, installed on a user’s PC (the PC usually connected to the measurement device or used for data processing) under the Microsoft windows operation system. This client is a tool for managing protocols. It provides a number of features for designing, viewing and exporting protocols. The main screen of the program is the list of templates and protocols under a given hierarchy with context menu which provides an ability to add new items, delete, archive or edit existing.

The software allows to:

- Manage the protocols and templates (protocol manager).
- Design the templates using GUI (template designer).
- Fill the protocols (protocol generator).
- Visualize and automatically process the data (custom modules).

#### Protocol manager

Protocol manager is a main BioWes module, which provides commands for visualization, creation and modification of protocols and templates. The protocols and templates are viewed in the form of a list with the basic information (name, description, author, last modification date) under a specific hierarchy. The organization-based hierarchy and research-topics hierarchy is available. Other modules are executed through this one: they can add commands to the menu of the protocol manager and provide additional functionality. For example, the modules for data processing are executable from the menu of the protocol manager.

The protocol manager provides access to the database for other modules. This module uses client communication interface for the connection to the database and it is responsible for the connection live cycle. The other modules have the access to the created connection to the database and they can use a part of client communication interface for data upload and download through the services exported by the protocol manager.

#### Template designer

Template designer allows a user to create Templates using the common graphical components, such as text fields (for both plain and rich text), check boxes, drop down menus, images, hyper-links, etc. The user simply drags and drops the components from components list to the designing window, place it and resize depending on the purpose. Each component has a list of properties that are shown in properties window. The common property of each component is a unique descriptor, which identifies the component within the protocol template. Component can be in two states:

1. *Read only* It means that this component is filled by the user during the template creation and cannot be changed in protocol based on this template. This state can be used for creating of guides through the experiment. The researcher can create the experimental plan and the technician has to follow the instructions and report measured values.
2. *Read/write* Components can be filled during protocol creation. This state assumes user input.

The user can also decide whether a particular component is mandatory. A person creating the protocol can be required to insert some important element such as a method description, while others, such as comment, remains optional. If it is necessary, dependencies between the components can be included. This feature provides flexibility. Routine work automation can be achieved using custom plugins. In the case of templates, plugins can be used for filling of components (e.g. with information extracted from experimental device). In such instances, desired plugin should be specified in ‘external’ property of an element.

Each template (or protocol based on this template) is represented by several tabs. By default, ‘general information’ and ‘data files’ tabs can be used to add a number of custom tabs in order to divide template to logical parts (sample preparation, device settings, experimental procedures, etc.). The user can check how protocol based on this template would look like using ‘preview’ feature.

Another feature of the template designer is terminology support: the user can add the URL of file in OWL format and use the list of definitions during the creation of the template.

A user can also choose from several saving options. It is possible to export template data to a file or import it from file, save intermediate result to the database or finalize the template. Finalization means that the template is ‘locked’ and cannot be changed. The finalization workflow functions so that only finalized templates are valid for creation of the protocols. However, a user can clone a finalized template in order to change some data (add new element, fix misprint, etc.) and save it as a new template. Such a function is a part of so-called ‘evolution of knowledge’ used for backtracking the changes made to the protocols or templates. It also simplifies the design of a new protocol for similar experiments.

#### Protocol generator

Protocol generator is a module for creating the protocols out of templates. The same template can be used for creating several protocols of similar experiments, which increases the level of standardization. The module can also serve as a guide throughout the experiment if the protocol contains the information about execution of the experiment.

To create a new protocol, the user selects the template from the list of finalized templates. The template has to be finalized (locked for modifications) because it contains a prescription for the protocol visualization and definitions of data types for data entry.

‘Data files’ tab serves as data storage: the user can specify the file(s) or folder(s) with experimental data that needs to be uploaded to the database. If the protocol is shared, the user can see the list of files and folders attached to this protocol and download an entire repository or single files. Such behavior implements a light sharing: only metadata are shared, but it is still possible to share the whole dataset as well.

BioWes functionality can be extended using plugins. Plugin is a DLL file that contains a code implementing desired functionality. There are generally two kinds of plugins: one for automated filling the data and for data processing. The user can invoke plugins responsible for filling the data by pressing the ‘fill protocol’ button.

The protocol itself can be linked to the parent protocol. This mechanism is used for creation of data processing chain in the repository. The user can create one protocol for project description, the second protocol describes the data measurement and the third one describes processing of measured data. The link to the parent protocol can be defined during the creation of the protocol or during the modification.

The second way to create a link with another protocol is to use the ‘protocol link’ component. If the template contains this component then the user can select the protocol from the list. If the user clicks to the linked Protocol in the protocol generator, it is opened in a new tab. In this way redundancy in the protocols can be reduced. If the experiment needs some preparatory actions (cell preparation, mixing of cultivation medium) then it can be described in a separated protocol and linked to the protocol of the experiment.

After filling the fields and attaching experimental data, the user can save protocol (if it is not completed ) or finalize it (it will make protocol non-editable). This guarantees that all mandatory fields are completed, otherwise, finalization is not possible. Also, all values can be cleared and the Protocol cloned in order to create a new one using a different name and data. Protocols can be exported as PDF documents.

### Standardization

The philosophy of the BioWes system is to offer standardization to the user without the necessity to learn new things which is usually the main issue of the standardization usage. Therefore standardization is hidden to the user or it is offered as a list of standardized terms based on the standardization provided by one of the standardization portals [14–17].

#### Standardized terminology

The system is designed to support the approved terminology that is encapsulated in the OWL ontology files which are provided by several standardization portals (see "Background" section). The user can use the terms from the standard for a description of the experimental conditions (cell line, magnification, etc.) during the design of the template (empty protocol). The standardized terminology improves the clarity of the metadata and enables multidisciplinary cooperation. The link to the standard is defined in the description of the template and the it can be used to clarify the terms for the specific research area.

**Fig. 2:**
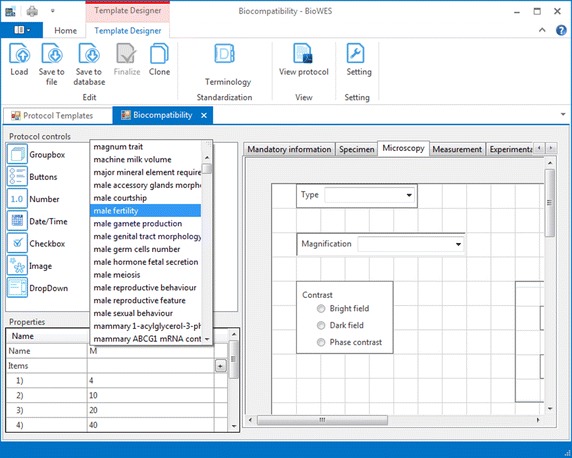
Example of the list of terms from the standard loaded from one of the standardization portals. The terms list is offered to the user during the design of the protocol template. Once the user start to define the trait (description of experimental conditions) the system provides the list of terms

**Fig. 3:**
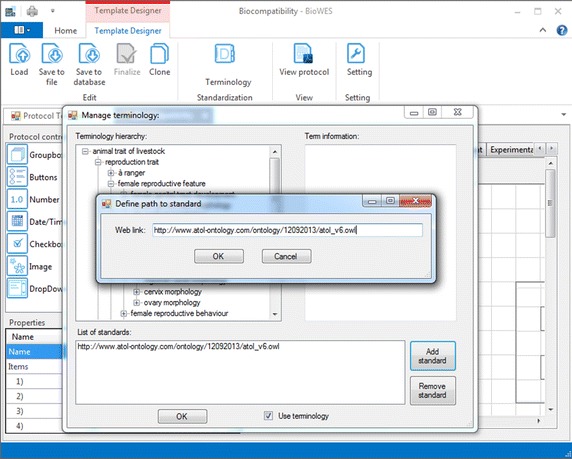
Dialog for the definition of the web link to the OWL file containing the standardized terminology

The standardized terminology is needed for the proper definition of the experimental protocol for the clarity of the individual experimental conditions. Protocol designer (tool for graphical design of the protocol template) enables the user to download the OWL file with the standardization from one of the standardization portals. The user provides the link to the OWL file storage and the system uploads the file to a local computer (see Figs. 2, 3). The terminology is extracted from the file and stored into the local terminology list of the template. A user can combine terminology from other standardization files. Information about standardization source is added into the description of the template. Any user who uses the template is acquainted with the terminology used for the particular template.

The terminology list is provided to the user in the form of whisperer during the design of the protocol. The terminology is used for the correct definition of individual components naming to describe the experimental condition. The user can decide to use his own terminology or select one of the terms from the list.

#### Standardized protocol structure description

**Fig. 4:**
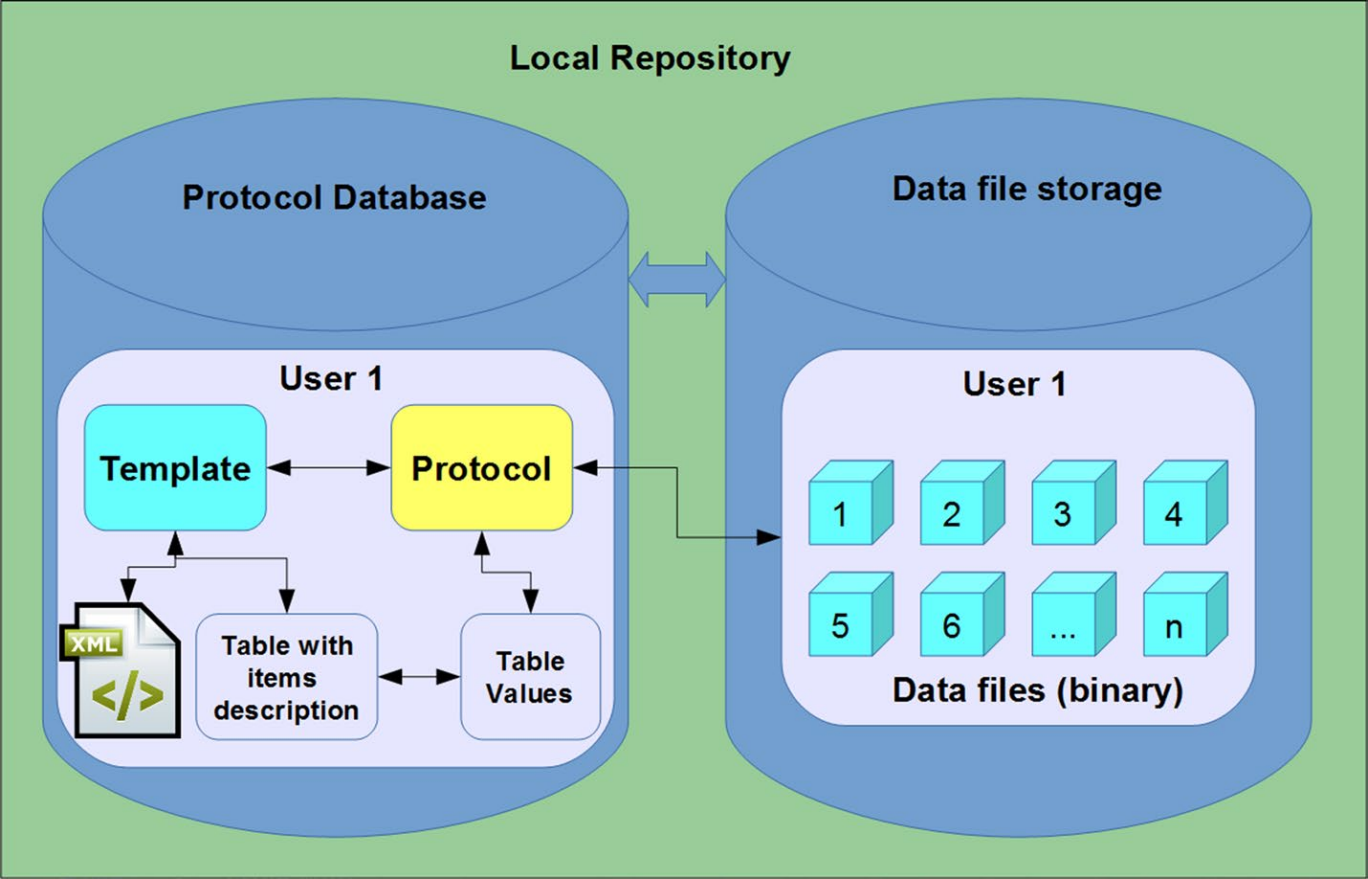
The scheme of the protocol template and protocol definition. Protocol template, protocol and data files are stored in Local repository. The Database for protocols and protocol templates is separated from the data files storage. Protocol template under User 1 account consists from the XML file with template appearance description and table with the list of the template items. Protocol under user1 account was derived from the protocol template. Protocol contains the list of protocol values corresponding to the list of protocol template items and the links to the individual data files stored in data file storage under user1 account

The basic unit of the BioWes system is the template (empty protocol), which defines protocol layout, template items (experiment description), protocol data types and protocol actions. Standardization of this set is needed to provide the possibility of searches, opened interface for Protocol visualization and protocol expert to another formats (PDF, XLS). To ensure the standardized description of the protocol, the graphical user interface for template design is provided to the user with predefined components. The template layout, components and protocol actions (events defined for buttons and check boxes) are stored in the XML file with defined structure for validation possibility. The items and items data types are stored directly in the database in the form of defined structure. This concept provides an option to change the template layout independently on the template values. The open interface is defined for access to the template. Therefore the users can implement their own software for the visualization of the template and trotocol. The protocol itself (filled template) consists of the tree separate entities: template, protocol values, and protocol files (see Fig. 4). Each protocol is based on the specific template, which is selected by the user. The template defines the Protocol layout and the items for protocol visualization. The particular values of the protocol items selected by the user are stored separately in the database to be independent on the Template. The data files are then stored in the separate repository (file system, database, cloud, etc.) with the direct link to the protocol. This concept enables independent manipulation with the protocol layout, protocol values and protocol data files. The user can use a third party software through the open interface to modify the protocol based on the specific access level. The standardized way of the template and protocol description improves the exchange of the protocol and enables the use of a third party software, which increase the usability of the system.

#### Standardized processing chain description

Data processing is a very important step of the data management systems, which is supported occasionally. Almost every experimental data set requires processing, which typically leads to the removal of redundant information and analysis of the remaining information content. This processing chain can consist of many processing steps using different methods and software. The global overview of the processing chain is very useful for later analysis of the research work. This process of keeping detailed information about data processing, in practice is unfortunately often underestimated and can lead to misinterpretation of results. Metadata used to describe the post-processing contain information about the original data (this information is a reference to the original data in the data storage area) and basic information on the method (software) processing and the setting (parameter setting software). Effective representation of this kind of metadata is a protocol processing experimental and its linkage to the protocol of the experiment original data. Therefore the BioWes system provides a mechanism for the linking of the protocols/templates and visualization of the protocol network. The links between protocols/templates are defined in the specialized table in the database. The record in the table contains the link to the parent object (protocol/template), child object, and the type of relation. The network of the protocols/templates simplify the orientation of the user in the individual processing steps and improves the possibility of more people collaboration.

### Custom plugins and modules

Functionality of BioWes desktop application can be extended using custom plugins for data extraction and modules for data processing based on BioWes SDK available for developers. Using the plugins and modules allows a user to automate such tasks as:

1. Filling the protocol with metadata extracted from measurement devices (e.g. microscope settings, image EXIF data, etc.).
2. Processing the experimental data attached to the Protocol (e.g. statistical analysis, post-processing and enhancement, etc.).
3. Visualization of experimental data.

From the technical point of view, plugins and modules are the DLL files executable by the Protocol Manager. They can be written in any programming language, as long as it’s possible to compile it to DLL files. Data extraction plugins are invoked during the Protocol creation and rely on unique ID of each Protocol field (in order to fill it properly). Data processing and visualization modules work with experimental data attached to the Protocol.

Using plugins will require less time on formal work such as filling out the Protocol and focus on research goals.

### Web interface

Web Interface allows a user to access the repository without installing the desktop Client. It provides a tool for managing protocols and templates stored in the database. It is implemented as a web application based on Apache web server and written in PHP and JavaScript programming languages. The main features are:

- Registering new user;
- viewing the list of protocols and templates (both owned and shared);
- viewing the metadata;
- viewing the content of the attached experimental data and downloading the data;
- sharing under different access right;
- full text search in the metadata of protocols and templates;
- visualization of the links between items for the possibility to backtrack the data processing steps in the protocols or analyzing the evolution of the template.

#### Users

Currently only the BioWes web interface is dealing with users.

User management consists of the two parts: registration and sharing-related activities. A new user can be registered via Web Interface: the newcomer should provide his or her email address, name and generate a password. It is also possible to retrieve a lost password of an existing user. The registration process can be extended with the confirmation step when the defined authority can accept or decline new registrations.

Sharing also implies the work with the list of users. BioWes item (protocol or template) can be shared with several users or with a user group (same access level is granted for each member of the group). The groups can be customized.

#### Filtering

The user can specify the number of tags for each protocol and template, which can later be used to filter the list of items. It works in the way that the user can narrow down the list by entering the tags of interest in ‘filtering options’ tab.

#### Sharing

Web interface allows users to share the protocols or templates with other registered users or user groups having different access rights.

The system provides eight levels of sharing so the user can restrict access to the data (protocols, templates and experimental data). Access levels are ascending: the higher level contains the access right of the lower level:

1. ‘Guest’ is the lower level. The user with ’Guest’ access can only read the general information;
2. ‘Reader’ access let the user read the meta-data;
3. ‘Follower’— read everything;
4. ‘Reviewer’ allows the user to modify general information;
5. ‘Contributor’ allows the user to modify general information;
6. ‘Master’— modify everything including experimental data;
7. ‘Co-author’— modify everything and re-share with access level up to ’Master’. It’s the highest accessible level;
8. ‘Owner’ is a system unchangeable access level granted only to creator of the protocol or template who has an exclusive right to delete items;

The 1–3 access levels (from ‘Guest’ to ‘Follower’) are read only levels: the user can see the information but cannot modify it. The 4–8 levels (from ‘Reviewer’ to ‘Owner’) are read/write levels: the user can modify information.

Re-sharing is possible starting from ‘Reader’ access level. The users with access level from ‘Reader’ to ‘Master’ can re-share the protocol or template to 3rd users, but only with ‘Guest’ access. On the other hand, a ‘Co-author’ user can re-share item with every access level except for the ‘Co-author’. Such behavior is designed to grant the author of the protocol an ability to maintain full control over sharing of sensitive data and, at the same time, restrict unauthorized re-sharing.

### Mobile interface

Mobile Interface is intended to serve as a system access point to those who require greater level of mobility. This interface allows researcher to fill the protocol or review it (e.g. in order to reproduce the experiment) ‘on-the-go’ using the mobile device—smartphone or tablet. From the technical point of view, Mobile Interface is a mobile oriented web service. That makes it cross-platform, independent on device’s operating system. Mobile interface can be used to perform field experiments or allow the technicians to follow the experiment step-by-step under specific process conditions (e.g. fish tanks) based on predefined protocol.

Currently mobile interface allows one to create the protocol, fill it with metadata and upload experimental data from the device. Template creation is not supported at the moment, templates should be created in advance in desktop client. User can perform the simple search using the protocol name and simple filters (all protocols / my protocols, finalized/not finalized).

### Protocols evolution

The BioWes system offers the possibility to link protocols using the connection between the parent protocol and child protocol. The user can define the link between protocols during the creation of a new protocol. The list of all protocols available to the user is shown in dialog window and the user can select a protocols as parent protocol of the currently created protocol. The list of parent protocols can be later modified in the protocol settings. The system of protocol links is used to describe two basic types of protocol relationships.

**Fig. 5:**
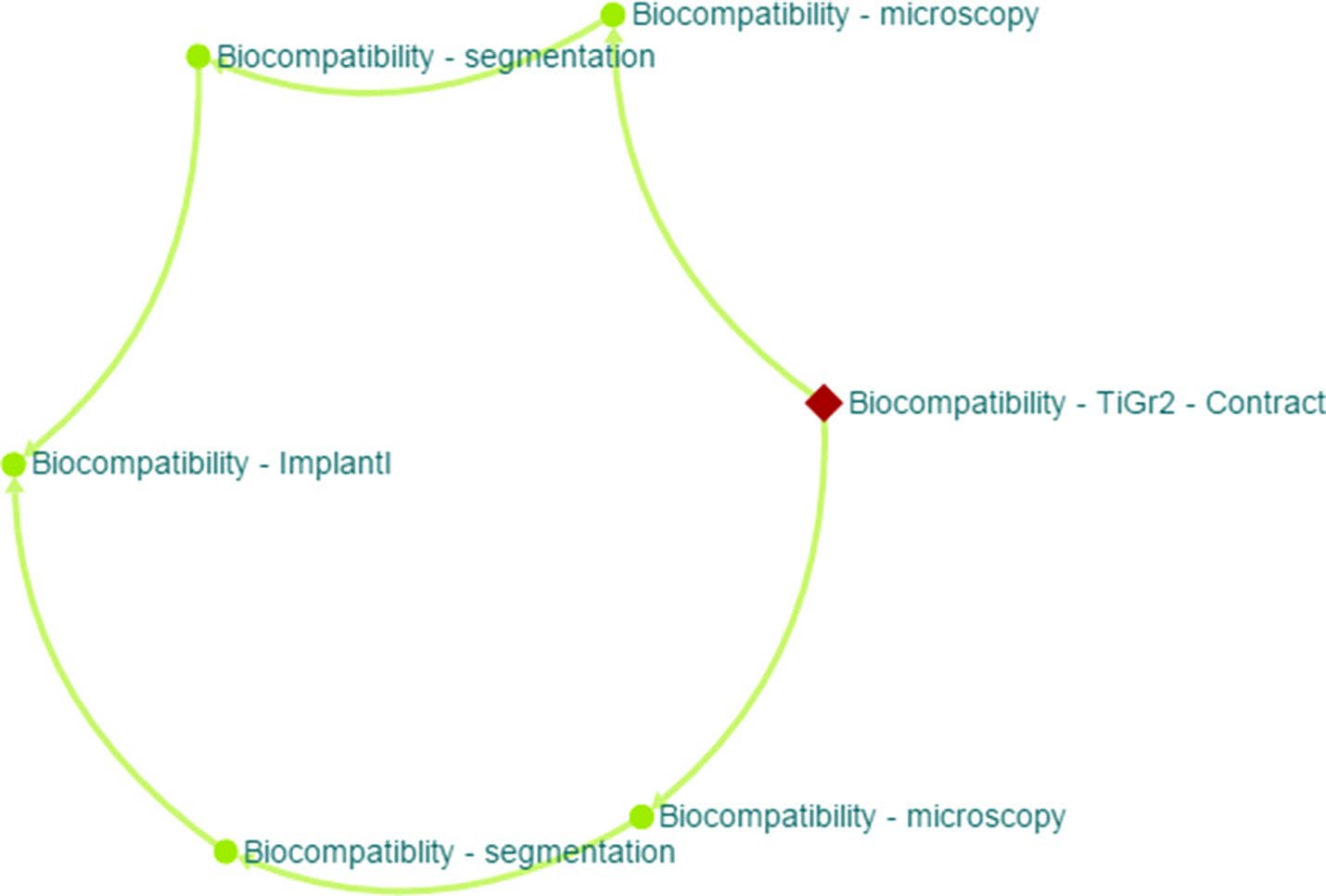
Example of the protocol processing chain visualization. Protocol biocompatibility—TiGr2—contract is the protocol with the definition of the work (tested material, types of test), protocol biocompatibility—microscopy describes the two different time-lapse microscopy experiments where image time series were recorded, protocols biocompatibility—segmentation describes the process of automatic cell colony detection in the time series and biocompatibility—implantl contains the analysis of the sample coverage by cell and decision about the biocompatibility level of the tested material.

The first relationship is the processing chain of the experimental data. The relation between protocols describes the individual processing steps the user has performed from the beginning until the final results. An example of a processing chain is shown in Fig. 5. The example describes the analysis of new material biocompatibility using time-lapse microscopy of the living tissue culture. Individual Protocols describe the specific conditions of the steps (overall process description, data acquisition, data processing, data analysis). Visualization of the Protocol chain provides complete overview of the work and enable the user to get the metadata and data of individual protocol.

The second type of relationship is the link between template and its clones. This type of template relationship enables the user to analyze the evolution of the description of particular experiment. Evolution of the experiment description can provide important information about changes of the convention of experimental work. Changes can be caused by evolution of the measurement devices, procedures or simply habits of technicians.

## Results and discussion

The 2011 survey on experimental data management showed that for most systems there is a lack of infrastructure for the daily usage by the researcher. The survey is available in Czech language only (http://www.biowes.org/survey). Participants in the survey included the research institutes (EU-13, non EU-3) and private companies (1) (University of South Bohemia (Czech), Institute of marine research (Netherlands), Cell Physiology & Scientific Film (Austria), IMARES (Netherlands), University of Lleida (Spain),Universidad Politecnica de Valencia (Spain), Istituto de Acuicultura de Torre de la Sal (Spain), INRA (France), NOFIMA (Norway), Wageningen University (Netherlands), IFREMER (France), Universidad Autónoma del Estado de Morelos (Mexico), SINTEF (Norway), Universidad de Guanajuato (Mexico), Centro de Investigación y Asistencia en Tecnología y Diseño del Estado de Jalisco (Mexico), Institute of Physical biology (Czech Republic), Prague fertility center (Czech Republic). The survey was included a detailed questionnaire and personal visits to the institutes. The information about the data management process was divided into four main parts: protocol design, standardization, data and metadata storage, data and metadata management tools. The survey showed that the researchers can create an adequate experimental description to ensure reproducibility of experiments. If some standardization is used then the laboratory standards (experience terminology, metadata formats, naming convention) only is used. The data and metadata are stored in personal computers or in a central server into user managed directories without any approved rules. The tools used for data management are usually simple full text search for query of specific data or protocol and ftp or email for the sharing with the cooperators.

Several issues were identified relative to common methodology and software tools used by the institutes. The approaches do not allow control of data flow, do not provide simple standardization support, the overview of experimental work by the supervision is very limited, there is a possibility of data loss, , and control over cooperation through sharing is complicated. No record of software for experimental data management was found at the institutes. However, IMARES and NOFIMA had experience with the sharing of experiment description and experimental data using specialized web based systems for marine data collection.

The software (LIMS, e-notebook) and platforms (SEEK, ELIXIR) described in the introduction section provide different possibilities for experimental data management and processing. The main disadvantage of the solutions is that they offer only partial support to the investigator for data and metadata management and limited standardization tools.

Platforms like SEEK or ELIXIR begin with the pre-existing data and provide the possibilities of data processing and sharing (offering data to the research community with limited access level control). The LIMS and e-notebook systems can be used for data management but the tools for support of standardization at different stages is missing.

The main advantages of the BioWes system is simple support of standardized terminology, standardized process of protocol design, backtracking of the data processing steps and protocol evolution or linkage between experiment description and experimental data. The modularity of the system and the black box data representation enables the use of the system for different areas of experimental research because the protocol can be designed by the user based on the specific needs and unlimited support of the data formats.

The use of standardized terminology is complicated in many fields of the research. The existence of the ontology portals [14–17] where the terminology from different research fields is collected in the form of ontology is just one of the assumptions to successfully use the terminology. The outcome of the survey highlighted that many laboratories use the standards developed just in the particular laboratory. Therefore, BioWes provides the possibility to use existing standardized terminology which is available on the standardization portals. The researcher need not to learn the terminology but can simply search for similar terms in the list. In this way, a user can still apply ordinary terms from their laboratory or switch to the term from the list. The BioWes platform already has five main pilot installations at different institutes dealing with completely different experiments and data types.

The complete solution is tested on experimental data analysis in the field of live cell microscopy and aquaculture systems object behavior under the Institute of Complex systems (Nove Hrady, Czech Republic). The particular plugin for communication with the microscope was developed to manage the experimental work of Laboratory of tissue culture specialized on the research contracts of time lapse microscopy biocompatibility analysis. Specific application examples include the description of experiments related to aquatic animals behavior, image processing experiments (changing of fish coloration, disease detection, etc.) and experiments aiming to test new approaches to information theory (Belousov–Zhabotinsky reaction, mathematical modeling of pattern formations).

The data processing module for the LC-MS data analysis and visualization was developed for the Institute of Microbiology, The Czech Academy of Sciences (Trebon, Czech Republic).

The standardized terminology developed in the frame of the AQUAEXCEL project is used for protocol design of the aquaculture experiments at French Research Institute for Exploration of the Sea (Issy-les-Moulineaux, France). It is used to support genetically oriented experiments with sea bass in re-circulation tanks.

An example of protocol created using the system can be found in supplementary materials.

The platform is also the official data storage system of the international infrastructure project AQUAEXCEL. The follow up project AQUAEXCEL 2020 will use the platform as the data management system for investigations from the Transnational access research subprojects realized at 19 different aquaculture research infrastructures.

The pilot installations have produced several templates for specific research areas tested under actual laboratory conditions. The particular examples for the time-lapse microscopy, fish coloration analysis or cell detection in microscopy image are available to the public under the testing account of the easily accessible local repository (see "Availability" section).

Examples of the data processing modules for the particular data were developed and are available at the BioWes platform web page. Two visualization modules and three data processing modules are available for the following topics: Fish tracking in tanks, Entropy module for image enhancement, Image representation, object labeling, Fish mortality data visualization and LC-MS data visualization.

The BioWes solutions deals with the experimental data as a black box capable of storing any kind of data acquired during different experiments. A disadvantage of the approach is that the system cannot use the data itself for the user queries. Searching of the particular protocol is based only on the description of the experiment (metadata), which is sufficient if the experiment is described well (all important variables of the experiment from the reproducibility point of view are described). The modules specialized for particular data can be used to mine the information from the experimental data and introduce it to the system.

The system supports all the data types and has a powerful flexible tool for protocol design. It makes BioWes applicable for wide range of scientific topics, system is not limited to a specific domain.

### Future development

Continual testing of the BioWes identified the way for possible future improvements, which will increase the utility of the system and platform independence in the near future:

- *Template versioning* will introduce a new approach to evolution of knowledge. This feature will allow keeping history of changes made to Template in one place rather than in several slightly different Templates. In this way the user can view all the history divided by versions (specified using finalization).
- *Mobile devices support* will bring BioWes to smartphones and tablets allowing the users to interact with the system ‘on the go’.
- *Hierarchy of protocol and templates* will let the user organize items in the convenient way (owned or shared Protocols, Protocols divided by topic, etc.).
- *QR code recognition for mobile devices*. Each Protocol in BioWes has its own unique QR code, which can be printed and attached to items related to this Protocol (chemicals, devices, etc.). Scanning such QR code with mobile device will open the corresponding Protocol.

## Conclusions

This paper describes the BioWes platform for the experimental data and metadata management. The outcome will support researchers from the outset in formulating a Protocol design through all steps leading to cooperation through the sharing. The main advantages of the system is the support of the terminology standardization, evolution of protocols, protocols processing chain and modular solution of the system which mainly improves the reproducibility of the research work. A single researcher or research consortium can benefit from the platform through the modularity of the solution. The specialized plugins and modules improve the level of automation of the Protocol filling and uploading to the local repository. The central repository connected to the several local repositories serves as the public information point for the Protocols exchange and link provider to the experimental data.

The solution has several pilot installations and it is the official data storage of an international infrastructure project. The data processing and visualization modules were developed for the specific research projects. The users can simply test the solution using freely available database and software.

## Availability and requirements

- *Project name*: BioWes—Distributed, knowledge-based repository for large datasets for biology, food safety and other biologic applications (TA01010214)
- *Project home page*: http://www.biowes.org
- *Operating system*: Windows (protocol manager), cross-platform (web interface)
- *Programming languages:* C#, PHP, JavaScript
- *System requirements:*
  - Software (Web Interface):
    - Web browser: Google Chrome, Mozilla Firefox, Internet Explorer, Opera, Safari.
  - Software (Desktop client):
    - Microsoft Windows 7, 8, 8.1
    - .NET Framework 4.5 or higher
  - Hardware (Desktop client):
    - *CPU*: 64-bit dual-core processor, 1GHz
    - *RAM*: 4Gb
    - *HDD*: 100Mb free space

**Demo:** The users can use the public local BioWes repository in order to test the system. The test user account is prepared in the system with the following credentials:

- *User name*: test@test.cz
- *Password*: test

The user can log in to the account with the pre-prepared protocols, experimental data and processed data. The user can simply test the tools and functionality of the repository without the need of creation of the protocols and storage of user data.

Desktop client configured for work with public database is available to download here: http://www.biowes.org/biowes-client/

Public Web interface is available here: http://160.217.215.251/

Video guides through the system are available here: http://www.biowes.org/see-new-video-guides/

## Abbreviations

AQUAEXCEL: aquaculture infrastructures for excellence in European fish research
IFREMER: French Research Institute for Exploitation of the Sea
EOL: encyclopedia of Life
SQL: structured query language
XML: extensible markup language
GUI: graphical user interface
PC: personal computer
DLL: dynamic-link library
OWL: web ontology language
PDF: portable document format
XLS: excel binary file format
SDK: software development kit
EXIF: exchangeable image file format
QR: quick response code
URL: uniform resource locator
LC-MS: liquid chromatography - mass spectrometry
